# Genomic characterization and virulence of *Streptococcus suis* serotype 4 clonal complex 94 recovered from human and swine samples

**DOI:** 10.1371/journal.pone.0288840

**Published:** 2023-07-27

**Authors:** Rujirat Hatrongjit, Parichart Boueroy, Piroon Jenjaroenpun, Thidathip Wongsurawat, Nattakan Meekhanon, Peechanika Chopjitt, Han Zheng, Nahuel Fittipaldi, Sorujsiri Chareonsudjai, Mariela Segura, Marcelo Gottschalk, Anusak Kerdsin

**Affiliations:** 1 Faculty of Science and Engineering, Department of General Sciences, Kasetsart University Chalermphrakiat Sakon Nakhon Province Campus, Sakon Nakhon, Thailand; 2 Faculty of Public Health, Kasetsart University Chalermphrakiat Sakon Nakhon Province Campus, Sakon Nakhon, Thailand; 3 Faculty of Medicine Siriraj Hospital, Department of Research and Development, Division of Bioinformatics and Data Management for Research, Mahidol University, Bangkok, Thailand; 4 Faculty of Veterinary Technology, Kasetsart University, Bangkok, Thailand; 5 National Institute for Communicable Disease Control and Prevention, State Key Laboratory of Infectious Disease Prevention and Control, Collaborative Innovation Center for Diagnosis and Treatment of Infectious Diseases, Chinese Center for Disease Control and Prevention, Changping, Beijing, China; 6 Faculty of Veterinary Medicine, Research Group on Infectious Diseases in Production Animals (GREMIP), University of Montreal, Quebec, Canada; 7 Faculty of Medicine, Department of Microbiology, Khon Kaen University, Khon Kaen, Thailand; 8 Research and Diagnostic Center for Emerging infectious Diseases (RCEID), Khon Kaen University, Khon Kaen, Thailand; Universidade de Lisboa Faculdade de Medicina, PORTUGAL

## Abstract

*Streptococcus suis* is a zoonotic pathogen that causes invasive infections in humans and pigs. Herein, we performed genomic analysis of seven *S*. *suis* serotype 4 strains belonging to clonal complex (CC) 94 that were recovered from a human patient or from diseased and clinically healthy pigs. Genomic exploration and comparisons, as well as *in vitro* cytotoxicity tests, indicated that *S*. *suis* CC94 serotype 4 strains are potentially virulent. Genomic analysis revealed that all seven strains clustered within minimum core genome group 3 (MCG-3) and had a high number of virulence-associated genes similar to those of virulent serotype 2 strains. Cytotoxicity assays showed that both the human lung adenocarcinoma cell line and HeLa cells rapidly lost viability following incubation for 4 h with the strains at a concentration of 10^6^ bacterial cells. The human serotype 4 strain (ID36054) decreased cell viability profoundly and similarly to the control serotype 2 strain P1/7. In addition, strain ST1689 (ID34572), isolated from a clinically healthy pig, presented similar behaviour in an adenocarcinoma cell line and HeLa cells. The antimicrobial resistance genes *tet*(O) and *ermB* that confer resistance to tetracyclines, macrolides, and lincosamides were commonly found in the strains. However, aminoglycoside and streptothricin resistance genes were found only in certain strains in this study. Our results indicate that *S*. *suis* CC94 serotype 4 strains are potentially pathogenic and virulent and should be monitored.

## Introduction

*Streptococcus suis* is a zoonotic pathogen that causes invasive infections in humans and pigs [1]. Among 29 serotypes, strains of serotypes 2 and 14 are the most frequently recovered from human patients with *S*. *suis* infections, although the other serotypes have occasionally been isolated from humans (i.e., serotypes 4, 5, 7, 9, 16, 21, 24, and 31) [1–8]. The first human with meningitis caused by *S*. *suis* serotype 4 was described in 1988 in the Netherlands, while a second case occurred in 2018 in Thailand involving a septic patient [7,8]. No multilocus sequence typing (MLST) information is available for the Dutch case, but characterization of the Thai serotype 4 strain showed that it belonged to sequence type (ST) 94, the origin of clonal complex (CC) 94 [8]. In pigs, serotype 4 strains have been isolated from both clinically healthy and diseased pigs in China, Thailand, Sweden, and Canada [9–14].

Understanding the diversity of *S*. *suis* strains presents major challenges, and some strains may be pathogenic pathotypes that have the potential to cause diseases. However, the definition of virulent or pathogenic *S*. *suis* remains controversial. Currently, there are several methods that try to predict whether *S*. *suis* strains are pathogenic, including classical virulence-associated gene (VAG) profiles of *epf*, *mrp*, and *sly* [15]; MLST [16]; comparative genome hybridization (CGH) [17]; whole-genome sequencing (WGS) [18,19]; and minimum core genome (MCG) [20]. Of these described methods, classical VAG profiles have been used to predict the virulence or pathogenic potential of *S*. *suis* serotype 2 strains, whereas classical VAG profiles were less frequently found in *S*. *suis* types 1/2, 9, 7 and 3 recovered either from diseased or healthy pigs in European countries [15]. Previous studies have demonstrated the use of MLST and serotyping to identify potentially pathogenic *S*. *suis* isolates [12,16,21]. CGH could be used to classify the *S*. *suis* strains tested into the following three groups: (i) epidemic and highly virulent (E/HV group), which included ST1, ST6, ST7, and ST11 isolates; (ii) virulent (V group), containing ST81, ST13, ST56, ST87, ST308, ST54, and ST53 isolates; and (iii) intermediately or weakly virulent (I/WV group), containing isolates belonging to several STs recovered from nonhuman sources [17]. Seven MCG groups and ungroupable MCGs were classified in the *S*. *suis* isolates, especially MCG group 1, which included all the highly virulent isolates of ST1 and ST7 [20].

To date, the genomes of the *S*. *suis* serotype 4 reference strain 6407 (ST54, recovered from a diseased pig) and US strains (STs 17, 23, 94, 964, 977, and 981) have been sequenced [9,21]. A previous study revealed that US CC94 strains were predominantly pathogenic strains [21]. Herein, we describe the genomic comparative analysis of seven *S*. *suis* serotype 4 CC94 strains recovered from a human patient and from diseased and clinically healthy pigs in Thailand and characterize their virulence using an *in vitro* assay. This study provides insights into the genomic characteristics, putative virulence genes, genetic relationships, and pathogenic capacity of Thai *S*. *suis* serotype 4 CC94 strains.

## Materials and methods

### Bacterial strains, identification, and antimicrobial susceptibility

The seven *S*. *suis* serotype 4 strains used in the current study originate from the blood of a human patient with septicemia (*N* = 1, strain name ID36054, recovered in 2011) [8], from a diseased pig (*N* = 1, strain name TRG6, recovered from lung in 2014) and from asymptomatic pigs (*N* = 5, strain names ID34545, ID34553, ID34572, ID34693, and ID34704, recovered from tonsils of slaughtering pigs in 2011) [12]. Isolates were cultured on sheep blood agar plates, and DNA was extracted using ZymoBIOMICS DNA Kits (Zymo Research, CA, USA) following the manufacturer’s instructions. Confirmation of species and serotyping of all *S*. *suis* strains were performed using previously described PCR assays [22,23].

Susceptibility to penicillin was determined by the minimum inhibitory concentration (MIC) following the M100 (32^nd^ edn) Clinical and Laboratory Standard Institute (CLSI-M100) guidelines [24] using the Liofilchem^®^ MIC Test Strip according to the manufacturer’s instructions (Liofilchem, Italy). We followed the standards defined in the 2022 CLSI-M100 guidelines to classify the penicillin susceptibility of the strains (MIC ≤0.12 μg/ml = susceptible; MIC 0.25–2 μg/ml = intermediate; MIC ≥4 μg/ml = resistant) [24]. Susceptibility to other antimicrobials, such as ceftriaxone, cefepime, azithromycin, erythromycin, tetracycline, clindamycin, levofloxacin, and chloramphenicol, was determined using the disk diffusion technique following the 2022 CLSI-M100 guideline [24]. Since there are currently no breakpoints recommended for *S*. *suis*, those for viridans group streptococci were used, as defined in the guidelines [24]. Based on the CLSI guidelines for viridans group streptococci, MIC testing to penicillin was performed given that CLSI guidelines do not provide zone diameter breakpoints for this antibiotic [24]. Therefore, we performed the MIC test strip procedure for penicillin and disc diffusion for the other antibiotics. *Streptococcus pneumoniae* strain ATCC 49619 was used as a control.

### Whole-genome sequencing

The genomes of all seven strains were sequenced using Illumina technology. Additionally, the genomes of three strains (ID36054, ID34572, and TRG6) were sequenced using Oxford Nanopore Technologies (ONT) as described elsewhere [25]. Briefly, Illumina sequencing libraries were generated using the NEBNext Ultra II DNA Library Prep Kit for Illumina (New England Biolabs, UK) following the manufacturer’s recommendations. The genomic DNA was randomly fragmented to a size of 350 bp, and the fragments were A-tailed and ligated with the adapter. Libraries were sequenced as paired-end reads (150 + 150 bp) using a HiSeq 2500 instrument. The sequencing adapters were trimmed using Fastp v0.19.5 (https://github.com/OpenGene/fastp), and the quality of clean reads was determined using FastQC v0.11.8 (https://www.bioinformatics.babraham.ac.uk/projects/fastqc/).

Library preparation for ONT sequencing followed the rapid barcoding DNA sequencing protocol of the SQK-RBK004 kit without DNA size selection (which preserves the plasmid DNA), and the libraries were sequenced using a single R9.4.1/FLO-MIN106 flow cell on a MinION Mk1B sequencer. We performed base calling and demultiplexed the raw data using Guppy v3.4.5 (ONT). The ONT adapters were trimmed using Porechop v0.2.4 (https://github.com/rrwick/Porechop). Quality control of ONT reads was carried out with Nanoplot v1.28.1 (https://github.com/wdecoster/NanoPlot). Hybrid assemblies with the ONT and Illumina data were generated using Unicycler v0.4.8 [26], and the genome sequences were checked for quality using QUAST v5.0.2 [27]. Genome sequences were submitted to the NCBI Prokaryotic Genome Annotation Pipeline (PGAP v4.12) for annotation. The default parameters were used for all software unless otherwise specified. The genome sequences of the seven *S*. *suis* serotype 4 strains were deposited in the NCBI GenBank under Bioproject accession number PRJNA691075 with GenBank accession numbers shown in Table 1.

**Table 1:** Genomic characteristics of seven *Streptococcus suis* serotype 4 clonal complex 94 isolates in this study.

Parameters	ID36054	TRG6	ID34572	ID34545	ID34553	ID34693	ID34704
Accession number	CP109939	CP109937-CP109938	CP109940	JAOXNE000000000	JAOXND000000000	JAOXNC000000000	JAOXNB000000000
Genome sequencing technology	ONT+Illumina	ONT+Illumina	ONT+Illumina	Illumina	Illumina	Illumina	Illumina
Source of isolation	Blood	Lung	Tonsil	Tonsil	Tonsil	Tonsil	Tonsil
Host	Human	Pneumonia pig	Asymptomatic pig	Asymptomatic pig	Asymptomatic pig	Asymptomatic pig	Asymptomatic pig
Year of isolation	2011	2014	2011	2011	2011	2011	2011
Location in Thailand	North, Lampang	Central, Nakhon Pathom	North, Phayao	North, Phayao	North, Phayao	North, Phayao	North, Phayao
Minimum core genome group (MCG)	MCG-3	MCG-3	MCG-3	MCG-3	MCG-3	MCG-3	MCG-3
Sequence type	ST94	ST94	ST1689	ST1689	ST1689	ST94	ST94
No. of contigs	1	2	1	70	138	71	98
Genome length (bp)	2,215,082	2,292,430 and 6,890	2,287,867	2,225,750	2,259,845	2,157,906	2,168,372
No. of coding sequences (CDS)	2,134	2,223	2,207	2,160	2,176	2,086	2,091
No. of tRNA	58	58	58	50	49	48	47
No. of rRNA	12	12	12				
Plasmid replicon type	None	Unknown	None	None	None	None	None
Antimicrobial resistance							
• Penicillin (MIC; mg/ml)	S (0.094)	S (0.032)	S (0.12)	S (0.12)	S (0.12)	S (0.047)	S (0.064)
• Ceftriaxone	S	S	S	S	S	S	S
• Cefepime	S	S	S	S	S	S	S
• Levofloxacin	S	S	S	S	S	S	S
• Chloramphenicol	S	S	S	S	S	S	S
• Tetracycline	R	R	R	R	R	R	R
• Erythromycin	R	R	R	R	R	R	R
• Azithromycin	R	R	R	R	R	R	R
• Clindamycin	R	R	R	R	R	R	R
Antimicrobial resistance genes							
• *tet(O)*	+	+	+	+	+	+	+
• *erm(B)*	+	+	+	+	+	+	+
• *ant(6)-Ia*	-	+	-	-	-	-	-
• *ant(9)-Ia*	-	-	+	+	+	-	-
• *aph(3’)-III*	-	+	-	-	-	-	-
• *lsa(E)*	-	-	+	+	+	-	-
• *lnu(B)*	-	-	+	+	+	-	-

### Bioinformatics analysis

Antimicrobial resistance genes were detected using ResFinder 4.1 [28]. Plasmid replicons were analysed using PlasmidFinder 2.1 and PLACNETw [29,30]. Sequence type (ST) was confirmed using the PubMLST database (https://pubmlst.org/organisms/streptococcus-suis). GoeBURST was used to analyse STs in CC94 [31]. Minimum core genome (MCG) sequence typing was performed according to a procedure described previously [20]. We used MyDbFinder 2.0, Center for Genomic Epidemiology, (https://cge.food.dtu.dk/services/MyDbFinder/), to screen the genomes of the serotype 4 strains for the presence of 99 virulence-associated genes (VAGs) that have been described as important for *S*. *suis* virulence or pathogenesis (S1 Table). The same approach was used to screen the genomes for the presence of two genes (G15: ATP-binding protein and G20: hypothetical protein) specific to human-associated clades (HAC) and pathogenic pathotype markers, including a copper-exporting ATPase 1, a type I restriction-modification system S protein, gene *SSU_RS03100* (hypothetical protein), gene *SSU_RS09155* (hypothetical protein), and gene *SSU-RS09525* (RNA-binding protein) [19,32,33]. Out of 99 virulence-associated genes (VAGs), the presence or absence of 22 VAGs that were described in a previous study [34] was determined using unweighted average linkage (UPGMA) with the DendroUPGMA program as described elsewhere [25].

A dataset containing 97 curated *S*. *suis* CC94 genomes [35] was used in combination with our seven serotype 4 genomes generated in this study to construct the phylogeny of the *S*. *suis* CC94 population (S2 Table). The phylogeny of the CC94 strains was determined using a reference genome-based single-nucleotide polymorphism (SNP) strategy with REALPHY [36]. The phylogenetic tree was visualized using iTOL V4 software [37]. *S*. *suis* serotype 2 strain P1/7 (accession no. CP003736) was used as the reference genome for SNP analysis.

Pangenome analyses were performed with the anvi’o v7 workflow [38]. This workflow identified gene clusters and single-copy genes in the study genomes, including three serotype 4 strains (ID34572, ID36054, and TRG6), two serotype 2 genomes of epidemic strain SC84 (accession no. FM252031) and the highly virulent strain P1/7 [17]. All genomes, in fastA format, were submitted for pangenome analysis using the ’anvi-run-workflow’ script. Genes were annotated using anvi-run-ncbi-cogs. All genomes were added to a new anvi’o genome storage using the ’anvi-gen-genomes-storage’ application. Then, the program ‘anvi-pan-genome’ was used to run pan-genomic analysis on all the stored genomes using NCBI’s blastp tool (https://blast.ncbi.nlm.nih.gov/Blast.cgi?PROGRAM=blastp&PAGE_TYPE=BlastSearch&LINK_LOC=blasthome). We used ’anvi-import-misc-data’ to import additional metadata and ’anvi-compute-genome-similarity’ to compute the average nucleotide identity (ANI) using the pyANI tool (https://github.com/widdowquinn/pyani). The pangenome was visualized in anvi’o using the ’anvi-display-pan’ application. The whole pangenome was divided into core and accessory bins based on gene cluster frequency. The UpSetR plot was generated using the UpSetR package [39] in the R program to visualize gene overlaps across bacterial strains. Specifically, gene lists from the pangenome results were prepared and input into the UpSetR package to generate the plots.

### Cell cytotoxicity assays

A human lung adenocarcinoma cell line (A549) and a human cervical cancer cell line (HeLa) were used to determine the cytotoxicity of four selected *S*. *suis* serotype 4 CC94 strains, including three ST94 strains (ID36054, ID34693, TRG6) and one ST1689 strain (ID34572). These two cell lines had previously been used to study interactions with *S*. *suis* serotype 2 strains [40]. These cell lines were purchased from the American Type Culture Collection (ATCC, MD, USA). Three ST94 strains were selected based on their representation of strains isolated from humans, diseased pigs and asymptomatic pigs, whereas the remaining strain was a representative ST1689 strain from asymptomatic pigs (Table 1). The serotype 2 ST1 strain P1/7 was used as a control. The A549 and HeLa cells were cultured in RPMI1640 (Gibco; Thermo Fisher Scientific) and DMEM (Gibco; Thermo Fisher Scientific), respectively, and supplemented with 10% foetal bovine serum, 100 U/ml of penicillin (Gibco; Thermo Fisher Scientific) and 100 mg/ml streptomycin (Gibco; Thermo Fisher Scientific). They were incubated at 37 °C with 5% CO_2_. All four strains of *S*. *suis* were cultured overnight on sheep blood agar at 37 °C with 5% CO_2_. *S*. *suis* inoculum was prepared in RPMI1640 or DMEM depending on cell types at concentrations of 1 × 10^3^, 1 × 10^4^, 1 × 10^5^ and 1 × 10^6^ CFU/ml. The human epithelial cells were infected with the *S*. *suis* P1/7 control strain and the *S*. *suis* serotype 4 strains at four concentrations for 2, 4, or 18 h, and subsequently, the effect of *S*. *suis* infection was determined using the CCK-8 assay (Merck, Darmstadt, Germany) according to the manufacturer’s instructions. This assay was performed in at least triplicate.

## Results and discussion

### MLST and MCG analysis

Hybrid Nanopore-Illumina assemblies allowed us to obtain high-quality genomes (1 or 2 contigs for two and one strains, respectively), while genome assemblies for the four strains sequenced by Illumina had only between 70 and 138 contigs. Comprehensive statistics for genome assemblies are provided in Table 1. Among the seven strains in the current study, only strain TRG6 from a diseased pig contained a plasmid (6,890 bp); however, the replicon type could not be identified by either PlasmidFinder 2.1 or PLACNETw (Table 1). This plasmid carried seven hypothetical protein genes, one *vanZ* family protein gene, and an unidentified replication protein gene.

**Fig 1:**
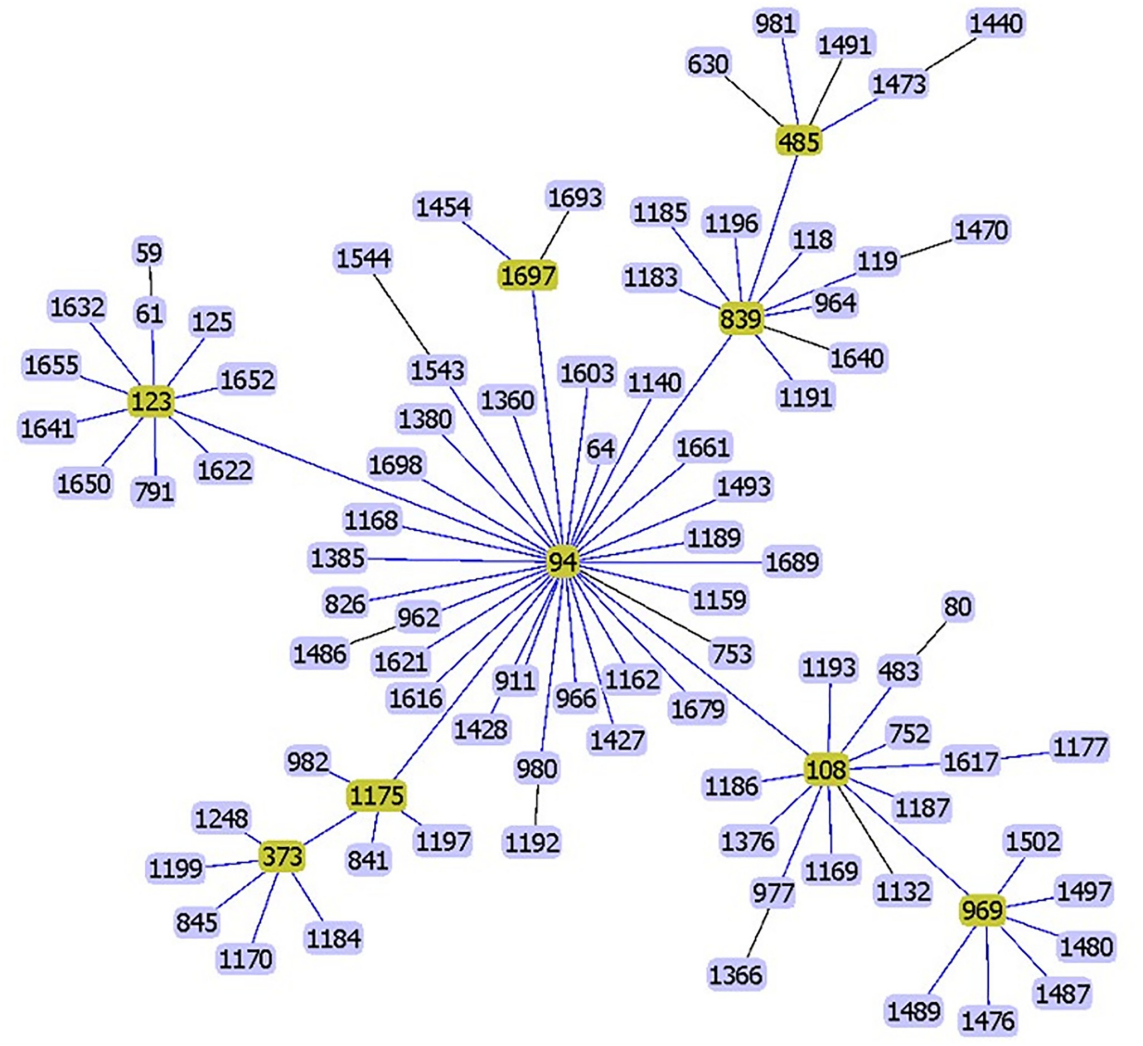
A goeBURST snapshot of sequence types in clonal complex 94. A total of 91 STs were included in this CC (access on February 10, 2023).

Genome-based MLST analysis of the seven *S*. *suis* serotype 4 strains confirmed that four strains were ST94 (ID36054, TRG6, ID34693 and ID34704) and three strains (ID34572, ID34545, ID34553) were ST1689; both STs were included among the CC94 strains (Table 1). As shown in Fig 1, CC94 is comprised of 91 STs based on sequences available in the PubMLST database as of Dec 9, 2022. ST1689 strains are a single allele variant of the *dpr* gene relative to ST94. A previous study demonstrated that CC1, CC28, CC94, and CC104 strains are associated with a pathogenic pathotype [21]. In CC94, STs 94, 108, and 977 were considered a “pathogenic pathotype” [21].

Analysis of MCG groups showed that all seven strains belonged to MCG group 3 (Table 1). MCG group 3 has been shown to include isolates from either diseased or clinically healthy pigs, which possess a higher number of virulence-associated genes than MCG groups 4 through 7, suggesting an increased potential for virulence [20].

### Antimicrobial resistance

As shown in Table 1, all seven serotype 4 strains were susceptible to penicillin, ceftriaxone, cefepime, levofloxacin, and chloramphenicol. Resistance to macrolides (erythromycin, azithromycin), tetracycline, and clindamycin (lincosamides) was detected in all tested strains. Worldwide, *S*. *suis* strains recovered from both humans and pigs have high resistance to tetracycline and moderate to high resistance to macrolides and lincosamides [41–48].

**Fig 2:**
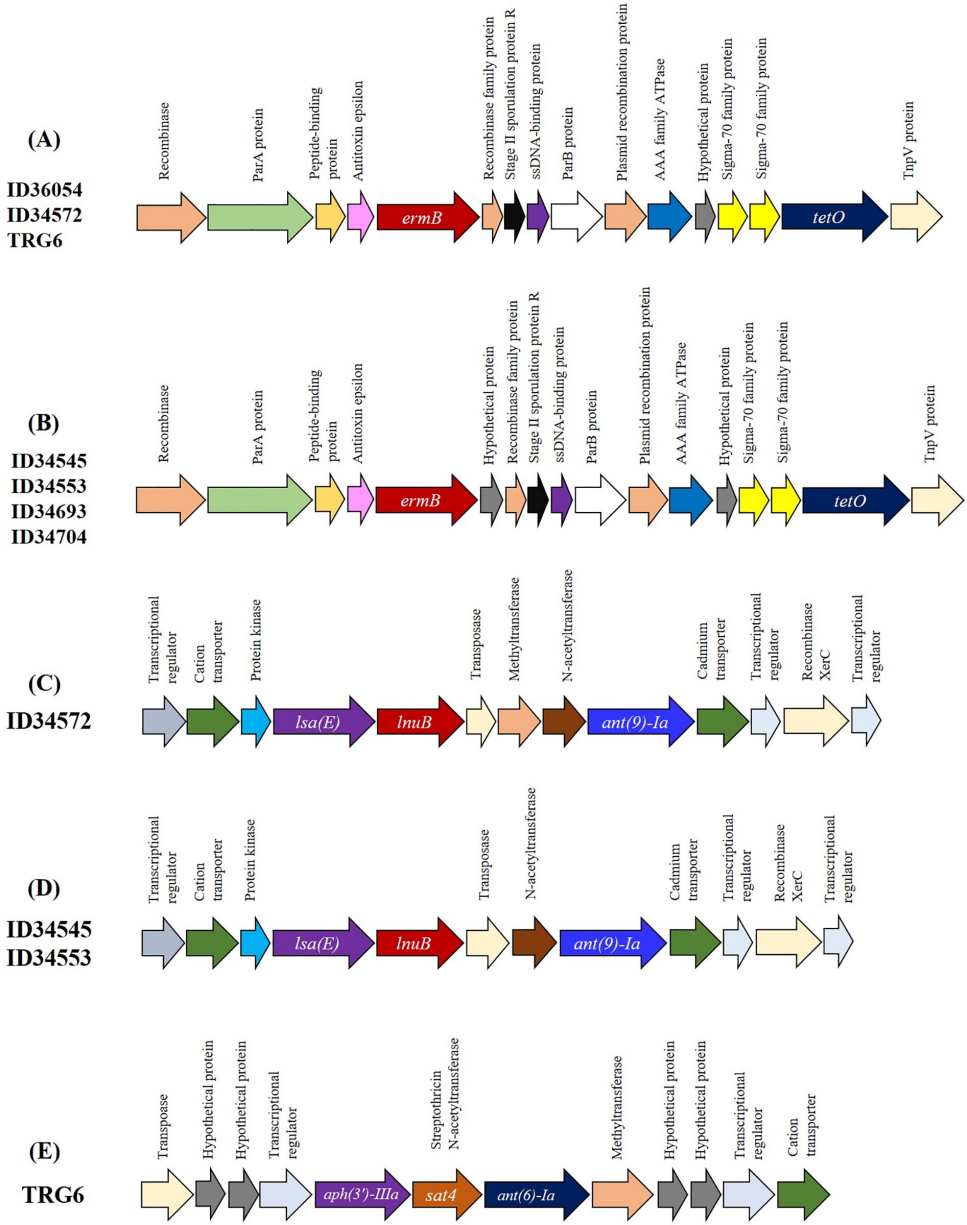
Genetic organization of antimicrobial resistance genes in all *Streptococcus suis* serotype 4 clonal complex 94 strains. A cluster of *ermB* and *tetO* was shown in strains ID36054, ID34572, TRG6, ID34545, ID34553, ID34693 and ID34704 (A and B). The hypothetical protein gene was present only in the *ermB-tetO* cluster in strains ID34545, ID34553, ID34693 and ID34704 (B). A cluster of *lsa(E)*, *lnuB* and *ant(9’)-Ia* was shown in strains ID34572, ID34545 and ID34553 (C and D). Methytransferase was absent in strains ID34545 and ID34553 (D). Strain TRG6 with the *aph(3’)-IIIa*, *sat4* and *ant(6’)-Ia* clusters (E).

ResFinder 4.1 identified the genes *tet*(O) and *erm*(B), which confer resistance to tetracycline and macrolide-lincosamide-streptogramin, respectively, in all strains under investigation (Fig 2). Several studies have shown that *tet*(O) and *erm*(B) are common among porcine and human *S*. *suis* isolates of various serotypes [43,48–52]. In addition, the genes *lsaE* and *lnuB*, which confer resistance to lincosamides, pleuromutilins and streptogramin A [48], were detected in all ST1689 strains (ID34572, ID34545, ID34553) (Fig 2). The genes *lsaE* and *lnuB* have been reported in human serotype 2 strains from Poland and pig strains of various serotypes from Vietnam, China, and the United Kingdom [49,51,53]. Two aminoglycoside resistance genes, *ant(6)-Ia* (aminoglycoside O-Nucleotidyltransferases) and *aph(3’)-III* (aminoglycoside 3’-phosphotransferases), were detected only in strain TRG6, which also possessed the streptothricin resistance gene (*sat4*) (Fig 2). The gene *ant(9)-Ia* (aminoglycoside nucleotidyltransferase), which confers aminoglycoside resistance, was detected in strains ID34572, ID34545, and ID34553. The prevalence of these antimicrobial resistance genes has been shown to vary among the different serotypes [51], but the *ant(6)-Ia* and *aph(3’)-III* genes have been documented in several porcine *S*. *suis* strains from Canada, China, Korea, and Thailand [41,51,52,54,55].

As shown in Fig 2, the genetic region carrying the *tet*(O) and *erm*(B) genes was similar among serotype 4 strains, the only difference being that a hypothetical protein gene downstream of *erm*(B) is found in strains ID34545, ID34553, ID34693, and ID34704, which is absent from the three other strains. The organization of the *tet*(O) and *erm*(B) genes is different in serotypes 4, 21, and 24 [25,56]. The cooccurrence of *erm*(B) and *tet*(O) has been reported in 69.06% (221/320) of *S*. *suis* from China and was also frequently detected in isolates from other countries [49,54–59].

It has been shown that some antimicrobial resistance genes are located on genomic islands integrated at the *rpsI* locus, examples of which are *ant(9)*-*erm*(B), *ant(9)-lnu*(B), *ant(9)*-*lnu*(C), *ant(9)*-*erm*(B)-*lnu*(B), *ant(9)*-*lnu*(C)-*erm*(B), *ant(9)*-*lnu*(B)*-lsa*(E), and *ant(9)*-*aph(3′)-IIIa*-*lnu*(B)*-lsa*(E)*-ant(6)*, which confer resistance to macrolides, lincosamides, and aminoglycosides [48]. Strains ID34572, ID34545, and ID34553 possessed the genes *ant(9)-Ia*-*lnu*(B)*-lsa*(E) arranged in a similar manner, while a methyltransferase gene was found in strain ID34572 (Fig 2). In strain TRG6, the *aph(3’)-III-sat4-ant(6)-Ia* cassette was similar to that reported in viridans streptococci, *S*. *pneumoniae*, *Enterococcus faecium* and *Enterococcus faecalis* [60,61].

### Virulence-associated genes

Analysis of 99 VAGs showed that 17 (*fhb*, *cbp40omp40*, *Hhly3*, *IgdE*, *neuB*, *NisK/NisR*, *pnuC*, *rgg*, *srtBCD* (*sbp1* and *sbp2*), *epf*, *nadR*, *revS*, *salK/salR*, *srtG*, *SSU05-0473*, *virB4* and *virD4*) were absent from all seven serotype 4 strains (S1 Table). The classical VAG profiles of the seven strains were *epf-/mrp+/sly+*. A previous study suggested that *epf*, *mrp*, and *sly* are mostly associated with serotype 2 and 14 strains, but they are very rare in other serotypes [15,62]. However, our data show that *mrp* and *sly* may be associated with CC94 serotype 4 strains.

A recent study revealed 21 potential zoonotic virulence factors (PZVFs), including *cbp40omp40*, *fhb-I*, *fhb-II*, *Hhly3*, *hyl*, *IdeS*, *IgdE*, *mrp*, *neuB*, *NisK*, *NisR*, *pnuC*, *rfeA*, *rgg*, *sbp1*, *sbp2*, *sly*, *sp1*, *tran*, *zmpC*, and *cps2BEFGJL* [35]. Note that these 21 PZVFs were mainly prevalent in CC1. Our seven serotype 4 CC94 strains showed nine PZVFs (42.8%), including *hyl*, *IdeS*, *zmpC*, *mrp*, *rfeA*, *sp1*, *sly*, *tran*, and *fhb-II* (S1 Table). The difference observed between the two studies may be explained by the fact that the PZVF in the previous study is more specific to CC1 than other CCs [35]. Genomic acquisition of different PZVFs may drive the emergence of a zoonotic clone [35]. In addition, CC94 may carry other PZVFs different from that of CC1; therefore, further study is necessary.

**Fig 3:**
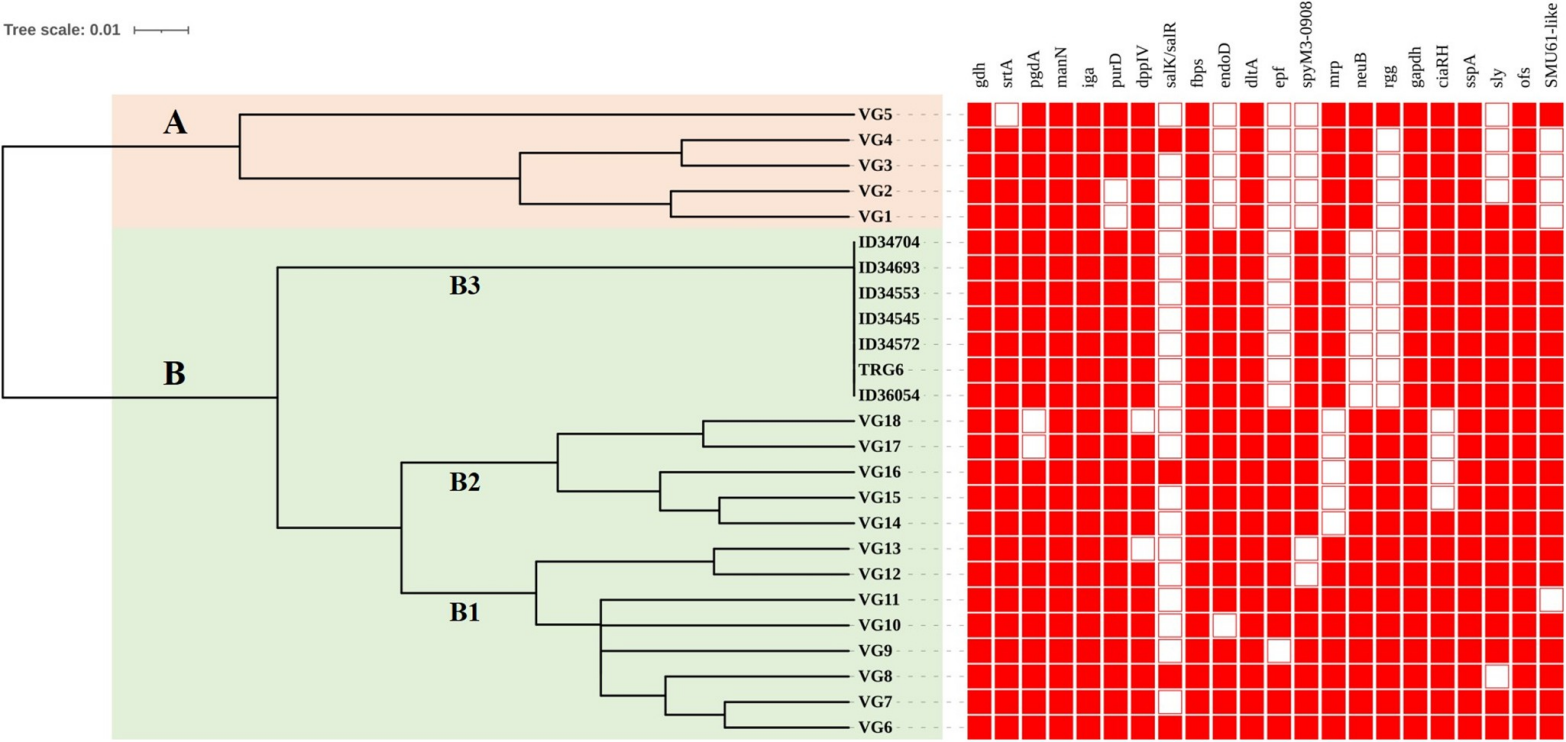
Clustering of *S*. *suis* serotype 4-CC94 strains based on the profiles of virulence-associated genes. VG is the virulence-associated gene profile. Filled squares refer to the presence of virulence-associated genes, and blank squares represent the absence of virulence-associated genes. Clusters A and B (B1-B3) are shown on the tree.

A previous study that analysed 22 VAGs (*gdh*, *srtA*, *pgdA*, *manN*, *iga*, *purD*, *dppIV*, *salK/R*, *fbps*, *endoD*, *dltA*, *epf*, *spyM3_0908*, *mrp*, *neuB*, *rgg*, *gapdh*, *ciaR/H*, *sspA*, *sly*, *ofs*, and *SMU_61-like)* identified 18 VAG profiles (VG1–VG18) among Chinese *S*. *suis* serotype 2 strains and divided them into two clusters A (VAGs 1–5) and B (VAGs 6–18) (S1 Table) [34]. Strains of cluster A lack six to eight of the 22 VAGs genes, while strains of cluster B normally possess six VAGs (*epf*, *sly*, *endoD*, *rgg*, *SMU_61-like*, and *SpyM3_0908*) that are rarely found in cluster A [34,63]. These six latter VAGs are often found among virulent serotype 2 strains [34]. Our analysis revealed three cluster B subclusters consisting of B1 (VG6-VG13), B2 (VG14-VG18), and the novel subcluster B3, which included all seven CC94 serotype 4 strains (Fig 3). Subcluster B1 lacked VAGs in the range of 1–3 genes, subcluster B2 lacked 2–5 VAGs, and subcluster B3 lacked four VAGs, including the genes *epf*, *neuB*, *rgg*, and *salK/salR*. Variation in the VAG distribution may be associated with the virulence of *S*. *suis*, as disease-associated *S*. *suis* strains seem to contain more virulence factors than non-disease associated strains [18], and studies using a zebrafish model have shown that strains of cluster B were virulent, whereas cluster A strains had relatively low virulence [34].

Compared with other studies, it seems that the putative virulence-associated genes have a differential distribution by *S*. *suis* lineages, implying that genes correlated with virulence may differ between lineages. Based on our analysis, serotype 4 CC94 strains carried many VAGs that might be considered potentially virulence, concordant with a previous study [21].

### Pathogenic pathotype determinants

Two previous studies have described markers of pathogenic pathotypes (or markers of disease-associated strains) [32,33]. These are a copper-exporting ATPase 1, a type I restriction-modification system S protein, the gene *SSU_RS03100* (hypothetical protein), the gene *SSU_RS09155* (hypothetical protein), and the gene *SSU-RS09525* (RNA-binding protein) [32,33]. Conversely, a putative sugar ATP-binding cassette transporter gene is a marker for non-pathogenic pathotype strains [32]. The CC94 serotype 4 strains in this study possessed all five pathogenic marker genes but lacked the non-pathogenic pathotype marker genes (S1 Table). This suggests that they may have virulence potential, which is consistent with the fact that two of the strains were isolated from an ill human patient and a diseased pig. The remaining five strains were isolated from clinically healthy pigs, which may indicate that clinically healthy pigs act as reservoirs of the pathogenic *S*. *suis* pathotype. A previous study demonstrated that *S*. *suis* strains with genotypes identical to pathogenic human strains were detected in asymptomatic healthy pigs [12]. In addition, CC94 was associated with pathogenic strains [21]. However, we cannot rule out that these pathogenic pathotype markers may fail to truly differentiate between non-pathogenic and pathogenic pathotypes, as is discussed elsewhere [64]. Further studies aimed at evaluating these five pathogenic pathotype markers in *S*. *suis* strains of different serotypes, sequence types, isolation sources, and geographic regions are required to better understand their usefulness as virulence predictors.

Dong et al proposed a panel of 25 marker genes as being strongly associated with human infections [19]. Among these, two genes (G15: ATP-binding protein and G20: hypothetical protein) were selected to be representative of the human-associated clade described in a previous study [19]. Analysis of these two HAC marker genes in our CC94 serotype 4 strains revealed that they were absent from the genomes of the strains, even though one of the strains was isolated from an ill human patient. It might be possible that these two HAC marker genes may be present in restricted *S*. *suis* populations or strains of some specific CCs. Therefore, more extensive analysis of these marker genes should be conducted to assess their capacity to predict whether a strain can cause human infections.

### Genomic comparison

**Fig 4:**
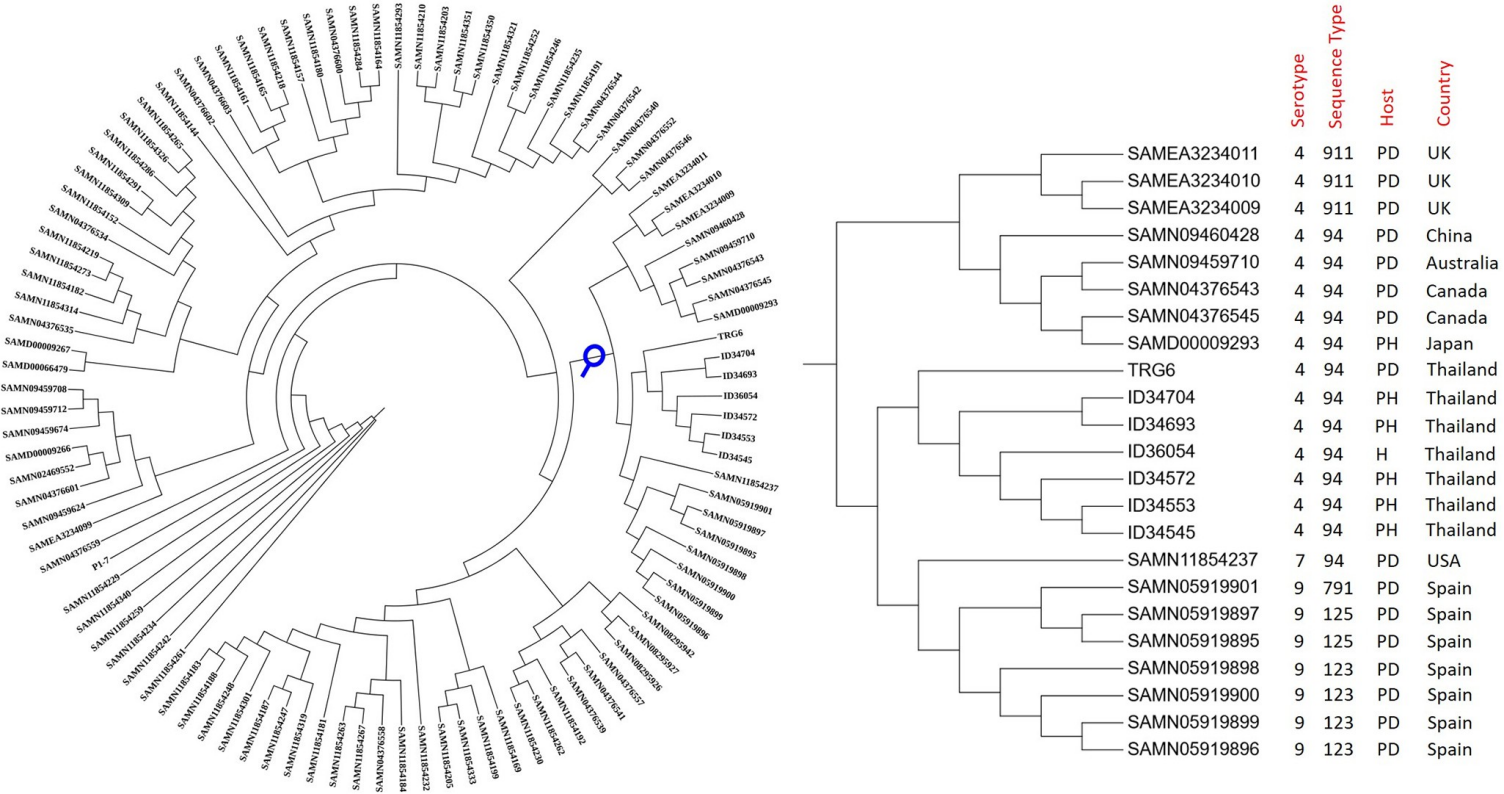
Whole-genome SNP reference mapping-based phylogeny of *S*. *suis* CC94 generated by CSI Phylogeny and visualized with the interactive tree of life tool. The clade of *S*. *suis* serotype 4 strains in the current study and their closely related strains are shown by the magnifying lens. PD, PH, and H were pig-diseased, pig-healthy, and human, respectively.

Fig 4 shows the whole-genome SNP reference mapping-based phylogeny of *S*. *suis* CC94; the phylogeny indicates that the CC94 serotype 4 strains under investigation clustered together with strains from Spain, including those of ST 123, 125 and 791, and a USA strain (ST94). These strains are of diseased pig origin and include strains of serotypes 7 and 9. A closely related clade to our serotype 4 clade contains serotype 4 strains from the UK (ST911), China (ST94), Australia (ST94), Canada (ST94), and Japan (ST94). All of these strains were of porcine origin.

**Fig 5:**
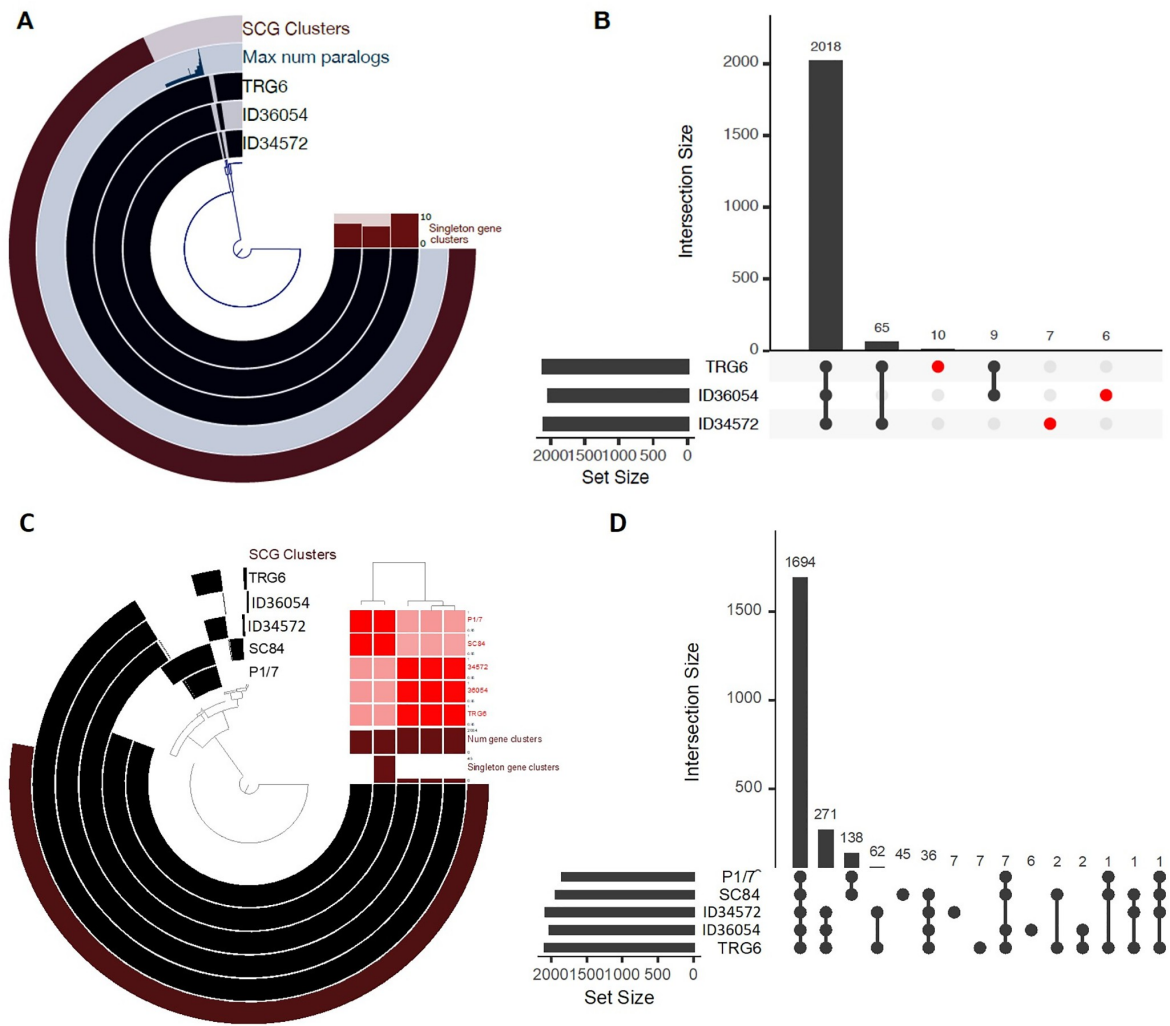
Pangenome analysis representation based on anvi’o software. (A) Pan-genome reconstructed with 3 complete genomes of *S*. *suis* serotype 4 strains. Each ring in the graph represents an individual *S*. *suis* genome, and each ray corresponds to a given gene homologue. The four inner layers are in black to designate gene clusters in that genome or translucent if the gene cluster is absent. (B) UpSetR showing the number of genes that are shared and unique between the three *S*. *suis* serotype 4 genomes. (C) Pan-genome reconstructed with 3 complete genomes of *S*. *suis* serotype 4 strains and 2 *S*. *suis* serotype 2 strains (P1/7 and SC84). Each ring in the graph represents an individual *S*. *suis* genome, and each ray corresponds to a given gene homologue. The four inner layers are in black to designate gene clusters in that genome or translucent if the gene cluster is absent. The ANI result was visualized in the heatmap tree. (D) UpSetR showing the number of genes that are shared and unique between the genomes of the serotype 4 and 2 strains.

We next compared the complete genomes of three serotype 4 strains, namely, strains ID36054 (ST94), TRG6 (ST94), and ID34572 (ST1689) (Fig 5A and 5B). A total of 2,018 coding sequences were found in these three strains. Strain TRG6 had 10 unique genes, while seven and six genes were unique to strains ID34572 and ID36054, respectively (Table 2; Fig 5B). TRG6 and ID34572 shared 65 genes not present in ID36054, while nine genes were shared between TRG6 and ID36054 but not ID34572. Table 2 shows a summary of the unique genes found in each of the three strains.

**Table 2:** Presentation of unique genes by comparison among three *Streptococcus suis* serotype 4 clonal complex 94 strains and two *Streptococcus suis* serotype 2 strains, SC84 (epidemic strain) and P1/7 (highly virulent strain).

Strain no.	Gene ID in S2 Table	GenBank database	% Identity	Representative strain	Country	Accession number
**ID36054**	6604	Hypothetical protein DP111_01800	100	*S*. *suis* serotype 4 strain SH1510	China	AXI64842
	6605	Hypothetical protein APQ97_05210	100	*S*. *suis* strain NSUI060	Canada	AML46483
	6611	No significant similarity found	-	-	-	-
	6615	Hypothetical protein	89.6	*S*. *suis* unknow strain	Unknow	WP_228477701
	6618	Tyrosine-type DNA invertase PsrA	100	*S*. *suis* unknow strain	China	WP_024408657
	6619	No significant similarity found	-	-	-	-
**ID34572**	6599	DNA recombinase	98.2	*S*. *suis* unknow strain	Unknow	WP_222290522
	6600	ABC-F type ribosomal protection protein Lsa(E)	100	*S*. *suis* unknow strain	Unknow	WP_257115242
	6607	Lincosamide nucleotidyltransferase Lnu(B)	100	*S*. *suis* unknow strain	Unknow	WP_170238913
	6610	Transposase	100	*S*. *suis* unknow strain	Unknow	WP_261309782
	6614	GNAT family N-acetyltransferase	100	*S*. *suis* strain 1481257	Canada	NQL74468
	6616	Uncharacterized protein	97.5	*S*. *suis* serotype 1 strain LSS99	UK	CYW37752
	6617	Aminoglycoside nucleotidyltransferase ANT(9)	100	*S*. *suis* strain NSUI002	Canada	ALA29506
**TRG6**	6597	Streptothricin N-acetyltransferase Sat4	99.4	*S*. *suis* unknow strain	Unknow	WP_258784539
	6598	RibD family protein	99.5	*S*. *suis* unknow strain	Unknow	WP_258784540
	6601	Single-stranded DNA-binding protein	98.7	*S*. *suis* unknow strain	Unknow	WP_222369822
	6602	HTH domain-containing protein	99.5	*S*. *suis* unknow strain	Unknow	WP_032533450
	6603	Nucleotidyltransferase domain-containing protein	100	*Clostridioides difficile* T5	Unknow	CCK89055
	6606	Putative membrane associated protein	100	*S*. *suis* serotype 2 strain GZ1	China	ADE31486
	6608	WYL domain-containing protein	99.1	*S*. *suis* unknow strain	Unknow	WP_014636359
	6609	Aminoglycoside 6-adenylyltransferase	99.7	*S*. *suis* unknow strain	Unknow	WP_170241231
	6612	IS1182-like element IS1182 family transposase	100	*S*. *suis* unknow strain	Unknow	WP_226315796
	6613	Aminoglycoside O-phosphotransferase APH(3’)-IIIa	100	*S*. *suis* unknow strain	Unknow	WP_264516777

We compared these three serotype 4 complete genomes with the representative genomes of serotype 2 epidemic strain SC84 (ST7) and serotype 2 highly virulent strain P1/7 (ST1). As shown in Fig 5C and 5D, the pangenomes of three serotype 4 and two serotype 2 strains revealed gene homologues and non-homologues. An ANI heatmap tree showed two clusters of gene contents (homologues and non-homologues) in serotype 4 and 2 strains (Fig 5C). A total of 1,694 genes were common between serotypes 4 and 2 strains, whereas 271 genes were present in the genomes of the serotype 4 strains but absent from the genomes of the representative virulent strains of serotype 2 (Fig 5D). Only 138 genes were unique to the serotype 2 strains, whereas 36 genes were shared between serotype 2 strain SC84 (ST7) and all three serotype 4 strains (Fig 5D). Each of the seven genes was unique to ID34572 and TRG6, whereas six unique genes were present in ID36054 (Fig 5D). Of note, a putative membrane-associated protein and aminoglycoside-6-adenylyltransferase genes were found in strain TRG6 and the two serotype 2 strains. Among the unique sequences present in the serotype 4 strains, we identified genes encoding antibiotic-resistance proteins (lincosamide, aminoglycoside, streptothricin), DNA processing proteins (single-strand binding protein, recombinase, invertase, transposases), domain-containing proteins (WYL, HTH, GNAT, RibD) and hypothetical proteins (Table 2).

### Cell cytotoxicity

**Fig 6:**
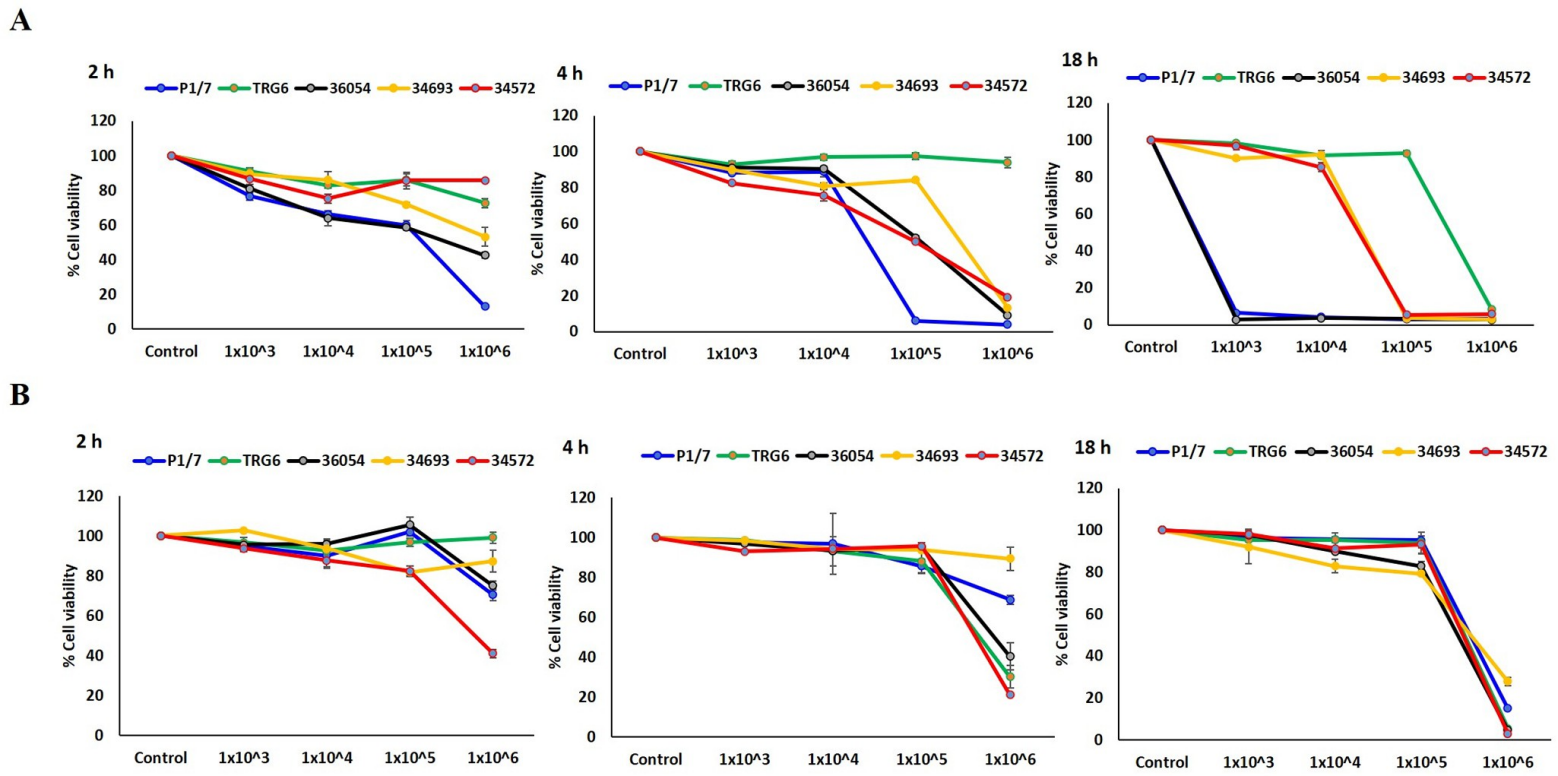
Cell cytotoxicity assay of three *S*. *suis* serotype 4 strains and control *S*. *suis* serotype 2 strain P1/7 on a human lung adenocarcinoma cell line (A549; A) and a human cervical cancer cell line (HeLa cell; B). Cell availability was determined according to time and dose. A549 cells showed higher susceptibility to *S*. *suis* serotype 4 and the control P1/7 strains than HeLa cells. At an infective dose of 1 x 10^6^ bacteria, both A549 and HeLa cells rapidly lost viability 4 h post-infection.

To begin to evaluate the virulence of serotype 4 CC94 strains, we chose three ST94 (TRG6, ID36054, ID34693) and one ST1689 (ID34572) strains, as they represented clinical cases from humans and pigs, as well as isolates from clinically healthy pigs. As shown in Fig 6, the A549 cell line showed higher susceptibility to *S*. *suis* serotype 4 and the control P1/7 strains than the HeLa cell line. At an infective dose of 1 x 10^6^ bacteria, both A549 and HeLa cells rapidly lost viability 4 h post-infection. Interestingly, cells infected with the human serotype 4 strain (ID36054) showed a decrease in cell viability comparable to that observed for the highly virulent control P1/7 strain, which contrasted with that of strain TRG6 (from a diseased pig). The ST1689 strain (ID34572) also induced rapid cell viability loss.

Because all tested strains in the current study carried suilysin (*sly*), the presence of suilysin is likely cytotoxic; this characteristic has been described elsewhere [65,66]. Several studies have shown that suilysin plays a role in pathogenesis; for example, suilysin induces membrane ruffling and uptake by epithelial cells by manipulating the host cell cytoskeleton [67], induces platelet aggregation [68], induces platelet-neutrophil complex formation [69], enhances blood‒brain barrier permeability by releasing arachidonic acid in brain microvascular endothelial cells [70], stimulates the release of heparin-binding protein from polymorphonuclear neutrophils [71], and induces TNFα release by monocytes [72].

The virulence of *S*. *suis* serotype 2 strains has been extensively characterized using both *in vitro* and *in vivo* models of infection; however, virulence studies of non-serotype 2 strains have only recently begun to be conducted. In one example, a *S*. *suis* serotype 31 strain (strain 11LB5) induced neurological symptoms in mice similar to those caused by a serotype 2 strain, while a *S*. *suis* serotype 28 (strain 11313) was non-virulent in mouse infection models [55]. An additional study showed that seven out of 47 *S*. *suis* serotype 31 strains isolated from clinically healthy pigs were also pathogenic in a zebrafish infection model [73]. *S*. *suis* serotype 8 strains were also proven to be virulent in mice and zebrafish [74]. A serotype 7-ST29 strain showed high survival in porcine blood that was obtained after the weaning of pigs and the strain caused meningitis and arthritis in an experimental infection of weaning piglets [75]. In addition, *S*. *suis* serotype 7 strains, including an MCG-3 strain isolated from a human patient, were lethal to experimentally infected mice [6]. Although we did not perform a full characterization of the virulence of serotype 4 CC94 strains, our cytotoxicity data together with our genetic findings suggest that some serotype 4 CC94 strains may be virulent. Further virulence studies, including in *in vivo* infection models, should be conducted to test this hypothesis.

## Conclusion

Genomic exploration and cytotoxicity tests of our *S*. *suis* serotype 4 CC94 strains isolated from patients, diseased pigs, and clinically healthy pigs revealed that they could be potentially virulent. They carried many virulence-associated genes often found in virulent serotype 2 strains and were cytotoxic to two cell lines. In addition to their potential pathogenicity, serotype 4 CC94 strains in the current study are carriers of the antimicrobial resistance genes *tet*(O) and *ermB*, which confer resistance to tetracycline, macrolides, and lincosamides.

## Supporting information

S1 Table Presentation of virulence-associated genes in *Streptococcus suis* serotype 4 strains. (XLSX)

S2 Table List of *Streptococcus suis* clonal complex 94 from GenBank. (XLSX)
